# Epidemiological, Clinical, and Laboratory Features of Children With COVID-19 in Turkey

**DOI:** 10.3389/fped.2021.631547

**Published:** 2021-05-07

**Authors:** Adem Karbuz, Gulsen Akkoc, Tugba Bedir Demirdag, Dilek Yilmaz Ciftdogan, Arife Ozer, Deniz Cakir, Selda Hancerli Torun, Eda Kepenekli, Tugba Erat, Nazan Dalgic, Sare Ilbay, Ayse Karaaslan, Emine H. Erdeniz, F. Deniz Aygun, S. Elmas Bozdemir, Nevin Hatipoglu, Melike Emiroglu, Zumrut Sahbudak Bal, Ergin Ciftci, Gulsum Iclal Bayhan, Zeynep Gokce Gayretli Aydin, Sevliya Ocal Demir, Omer Kilic, Mustafa Hacimustafaoglu, Dicle Sener Okur, Semra Sen, Aysun Yahsi, Hacer Akturk, Benhur Cetin, Murat Sutcu, Manolya Kara, Hatice Uygun, Tugce Tural Kara, Gulay Korukluoglu, Ozlem Akgun, Gülnihan Üstündağ, Mevsim Demir Mis, Enes Sali, Ozge Kaba, Nurhayat Yakut, Orhan Kılıc, M. Kemal Kanik, Ceren Cetin, Adem Dursun, Muharrem Cicek, Esra Kockuzu, Esra Sevketoglu, Gulsum Alkan, Gizem Guner Ozenen, Erdal İnce, Zekiye Baydar, Ahmet Kagan Ozkaya, Husnu Fahri Ovali, Seher Tekeli, Solmaz Celebi, Birgul Cubukcu, Alkan Bal, Fidan Khalilova, Mehmet Kose, Halil Ugur Hatipoglu, Tahir Dalkiran, Mehmet Turgut, Ayse Basak Altas, Hatice Nilgün Selcuk Duru, Ahu Aksay, Sevcan Saglam, Mehpare Sari Yanartas, Zeynep Ergenc, Yasemin Akin, Yeter Duzenli Kar, Sabit Sahin, Sadiye Kubra Tuteroz, Nimet Melis Bilen, Halil Ozdemir, Mine Cidem Senoglu, Burcu Pariltan Kucukalioglu, Gulser Esen Besli, Yalcin Kara, Cansu Turan, Burcu Selbest Demirtas, Aydın Celikyurt, Yasemin Cosgun, Murat Elevli, Aslihan Sahin, Serife Bahtiyar Oguz, Ayper Somer, Bulent Karadag, Recep Demirhan, Hatice Turk Dagi, Zafer Kurugol, Esra Cakmak Taskin, Aysegul Sahiner, Edanur Yesil, Yildiz Ekemen Keles, Remzi Sarikaya, Ela Erdem Eralp, Ferda Ozkinay, Hatice Kubra Konca, Songul Yilmaz, Yasemin Gokdemir, Gul Arga, Seval Ozen, Fevziye Coksuer, Goksel Vatansever, Hasan Tezer, Ates Kara

**Affiliations:** ^1^Division of Pediatric Infectious Diseases, Okmeydani Training and Research Hospital, University of Health Sciences, Istanbul, Turkey; ^2^Division of Pediatric Infectious Diseases, Istanbul Haseki Training and Research Hospital, University of Health Sciences, Istanbul, Turkey; ^3^Department of Pediatric Infectious Diseases, Faculty of Medicine, Gazi University, Ankara, Turkey; ^4^Department of Pediatric Infectious Diseases, Faculty of Medicine, Izmir Tepecik Training and Research Hospital, University of Health Sciences, Izmir, Turkey; ^5^Division of Pediatric Infectious Diseases, Van Training and Research Hospital, University of Health Sciences, Van, Turkey; ^6^Division of Pediatric Infectious Diseases, Umraniye Training and Research Hospital, University of Health Sciences, Istanbul, Turkey; ^7^Department of Pediatric Infectious Diseases, Faculty of Medicine, Istanbul University, Istanbul, Turkey; ^8^Division of Pediatric Infectious Diseases, Faculty of Medicine, Pendik Training and Research Hospital, Marmara University, Istanbul, Turkey; ^9^Division of Pediatric Infectious Diseases, Sanliurfa Training and Research Hospital, Şanlıurfa, Turkey; ^10^Division of Pediatric Infectious Diseases, Sisli Hamidiye Etfal Training and Research Hospital, University of Health Sciences, Istanbul, Turkey; ^11^Department of Pediatric Infectious Diseases, Faculty of Medicine, Hacettepe University, Ankara, Turkey; ^12^Division of Pediatric Infectious Diseases, Kartal Dr. Lutfi Kırdar Training and Research Hospital, University of Health Sciences, Istanbul, Turkey; ^13^Division of Pediatric Infectious Diseases, Erzurum Training and Research Hospital, Erzurum, Turkey; ^14^Division of Pediatric Infectious Diseases, Istanbul Kanuni Sultan Süleyman Training and Research Hospital, University of Health Sciences, Istanbul, Turkey; ^15^Division of Pediatric Infectious Diseases, Dortcelik Children's Training and Research Hospital, Bursa, Turkey; ^16^Division of Pediatric Infectious Diseases, Bakirkoy Sadi Konuk Training and Research Hospital, University of Health Sciences, Istanbul, Turkey; ^17^Department of Pediatric Infectious Diseases, Faculty of Medicine, Selcuk University, Konya, Turkey; ^18^Department of Pediatric Infectious Diseases, Faculty of Medicine, Ege University, Izmir, Turkey; ^19^Department of Pediatric Infectious Diseases, Faculty of Medicine, Ankara University, Ankara, Turkey; ^20^Department of Pediatric Infectious Diseases, Faculty of Medicine, Yildirim Beyazit University, Ankara, Turkey; ^21^Department of Pediatric Infectious Diseases, Faculty of Medicine, Farabi Hospital, Karadeniz Technical University, Trabzon, Turkey; ^22^Division of Pediatric Infectious Diseases, Goztepe Training and Research Hospital, Istanbul Medeniyet University, Istanbul, Turkey; ^23^Department of Pediatric Infectious Diseases, Faculty of Medicine, Eskisehir Osmangazi University, Eskisehir, Turkey; ^24^Department of Pediatric Infectious Diseases, Faculty of Medicine, Uludag University, Bursa, Turkey; ^25^Division of Pediatric Infectious Diseases, Denizli Hospital, Denizli, Turkey; ^26^Department of Pediatric Infectious Diseases, Faculty of Medicine, Celal Bayar University, Manisa, Turkey; ^27^Division of Pediatric Infectious Diseases, Samsun Training and Research Hospital, University of Health Sciences, Samsun, Turkey; ^28^Department of Pediatric Infectious Diseases, Faculty of Medicine, Koc University, Istanbul, Turkey; ^29^Department of Pediatric Infectious Diseases, Faculty of Medicine, Erciyes University, Kayseri, Turkey; ^30^Department of Pediatric Infectious Diseases, Faculty of Medicine, Istinye University, Istanbul, Turkey; ^31^Division of Pediatric Infectious Diseases, Necip Fazil Training and Research Hospital, University of Health Sciences, Kahramanmaraş, Turkey; ^32^Department of Pediatric Infectious Diseases, Faculty of Medicine, Adıyaman University, Adıyaman, Turkey; ^33^Department of Pediatric Infectious Diseases, Faculty of Medicine, Akdeniz University, Antalya, Turkey; ^34^Public Health Institutions of Turkey, Director of Virology Department, Ankara, Turkey; ^35^Division of Pediatrics, Istanbul Haseki Training and Research Hospital, University of Health Sciences, Istanbul, Turkey; ^36^Division of Pediatric Infectious Diseases, Izmir Tepecik Training and Research Hospital, University of Health Sciences, Izmir, Turkey; ^37^Van Training and Research Hospital, University of Health Sciences, Van, Turkey; ^38^Division of Pediatrics, Sanliurfa Training and Research Hospital, Şanlıurfa, Turkey; ^39^Division of Pediatrics, Erzurum Training and Research Hospital, Erzurum, Turkey; ^40^Division of Pediatrics, Istanbul Kanuni Sultan Süleyman Training and Research Hospital, University of Health Sciences, Istanbul, Turkey; ^41^Division of Pediatrics, Dortcelik Children's Training and Research Hospital, Bursa, Turkey; ^42^Division of Pediatric Intensive Care, Bakirkoy Sadi Konuk Training and Research Hospital, University of Health Sciences, Istanbul, Turkey; ^43^Department of Pediatrics, Faculty of Medicine, Yildirim Beyazit University, Ankara, Turkey; ^44^Department of Pediatric Emergency, Faculty of Medicine, Karadeniz Technical University, Trabzon, Turkey; ^45^Department of Pediatrics, Goztepe Training and Research Hospital, Istanbul Medeniyet University, Istanbul, Turkey; ^46^Department of Pediatrics, Faculty of Medicine, Eskisehir Osmangazi University, Eskişehir, Turkey; ^47^Denizli Hospital, Denizli, Turkey; ^48^Department of Pediatric Emergency, Faculty of Medicine, Manisa Celal Bayar University, Manisa, Turkey; ^49^Department of Pediatrics, Faculty of Medicine, Koc University, Istanbul, Turkey; ^50^Department of Pediatric Pulmonology, Faculty of Medicine, Erciyes University, Kayseri, Turkey; ^51^Department of Pediatrics, Faculty of Medicine, Istinye University, Istanbul, Turkey; ^52^Division of Pediatric Intensive Care, Necip Fazıl Training and Research Hospital, University of Health Sciences, Kahramanmaraş, Turkey; ^53^Division of Pediatrics, Kartal Dr. Lutfi Kırdar Training and Research Hospital, University of Health Sciences, Istanbul, Turkey; ^54^Department of Pediatrics, Kanuni Training and Research Hospital, University of Health Sciences, Trabzon, Turkey; ^55^Division of Pediatric Pulmonology, Pendik Training and Research Hospital, Marmara University, Istanbul, Turkey; ^56^Division of Thoracic Surgery, Kartal Dr. Lutfi Kırdar Training and Research Hospital, University of Health Sciences, Istanbul, Turkey; ^57^Department of Microbiology, Faculty of Medicine, Selcuk University, Konya, Turkey; ^58^Department of Pediatric Emergency, Faculty of Medicine, Ankara University, Ankara, Turkey

**Keywords:** COVID-19, pediatric, epidemiology, laboratory findings, disease severity

## Abstract

**Objectives:** The aim of this study is to identify the epidemiological, clinical, and laboratory features of coronavirus disease 2019 (COVID-19) in children.

**Methods:** A retrospective study was conducted by pediatric infectious disease specialists from 32 different hospitals from all over Turkey by case record forms. Pediatric cases who were diagnosed as COVID-19 between March 16, 2020, and June 15, 2020 were included. Case characteristics including age, sex, dates of disease onset and diagnosis, family, and contact information were recorded. Clinical data, including the duration and severity of symptoms, were also collected. Laboratory parameters like biochemical tests and complete blood count, chest X-ray, and chest computed tomography (CT) were determined.

**Results:** There were 1,156 confirmed pediatric COVID-19 cases. In total, male cases constituted 50.3% (*n* = 582) and females constituted 49.7% (*n* = 574). The median age of the confirmed cases was 10.75 years (4.5–14.6). Of the total cases, 90 were younger than 1 year of age (7.8%), 108 were 1–3 years of age (9.3%), 148 were 3–6 years of age (12.8%), 298 were 6–12 years of age (25.8%), 233 were 12–15 years of age (20.2%), and 268 cases were older than 15 years of age (23.2%). The most common symptom of the patients at the first visit was fever (50.4%) (*n* = 583) for a median of 2 days (IQR: 1–3 days). Fever was median at 38.4°C (38.0–38.7°C). The second most common symptom was cough (*n* = 543, 46.9%). The other common symptoms were sore throat (*n* = 143, 12.4%), myalgia (*n* = 141, 12.2%), dyspnea (*n* = 118, 10.2%), diarrhea (*n* = 112, 9.7%), stomachache (*n* = 71, 6.1%), and nasal discharge (*n* = 63, 5.4%). When patients were classified according to disease severity, 263 (22.7%) patients were asymptomatic, 668 (57.7%) patients had mild disease, 209 (18.1%) had moderate disease, and 16 (1.5%) cases had severe disease. One hundred and forty-nine (12.9%) cases had underlying diseases among the total cases; 56% of the patients who had severe disease had an underlying condition (*p* < 0.01). The need for hospitalization did not differ between patients who had an underlying condition and those who do not have (*p* = 0.38), but the need for intensive care was higher in patients who had an underlying condition (*p* < 0.01). Forty-seven (31.5%) of the cases having underlying conditions had asthma or lung disease (38 of them had asthma).

**Conclusions:** To the best of our knowledge, this is one of the largest pediatric data about confirmed COVID-19 cases. Children from all ages appear to be susceptible to COVID-19, and there is a significant difference in symptomatology and laboratory findings by means of age distribution.

## Introduction

At the end of January 2020, a new and unknown disease presenting with pneumonia and acute respiratory distress syndrome arose from China. In a few weeks, many different regions were affected globally, including the USA and Europe (1). The disease was named as coronavirus disease 2019 (COVID-19) because of the etiologic agent, which was a new coronavirus. By March 2020, COVID-19 was declared to reach a pandemic status (2). According to the latest data by the WHO, there are 98.3 million cumulative cases globally of which 4.1 million were reported by the end of January, and there are 2.1 million cumulative deaths of which 95,991 occurred by the end of January (3).

At the early stages of the outbreak, the disease was known to be more common among adults, and children were less susceptible to the disease and composed a very small part of the chart. Suggested reasons for this theory were having a more active innate immune response, healthier respiratory tracts (because of not being exposed to cigarette smoke and air pollution as compared with adults), virus–virus interaction and competition, age-related ACE-2 expression, and ADE mechanism with fewer underlying disorders (4, 5). Later on, the number of child infection cases has increased significantly. It is known that the majority of pediatric cases are asymptomatic or mild, but still some infected children may develop multisystem inflammatory syndrome (6).

While the incidence of MIS-C is uncertain, it appears to be a rare complication of COVID-19 in children.

Since then, it was confirmed that children were also at risk because of the difficulty in preventative and infection control measures (7). Besides, it was argued that children might become a spreader at the explosion phase of the outbreak (8).

On March 11, 2020, the first COVID-19 case was reported from Turkey, and cases increased gradually like in other parts of the world. Pediatric patients were also diagnosed and treated as much as adults. Despite the worldwide spread, the epidemiological and clinical patterns of COVID-19 in pediatric patients still remain largely unclear (9).

This report aims to identify the epidemiological characteristics and clinical findings in pediatric patients with the 2019 novel coronavirus disease in Turkey by May 1, 2020.

## Materials and Methods

### Data Acquisition

A retrospective study was conducted by pediatric infectious disease specialists from 32 different hospitals from all over Turkey by case record forms. Patients were diagnosed by pediatricians according to the guidelines developed by the Turkish Republic, Ministry of Health. Case definitions, either suspected or confirmed, (10).

Pediatric cases who were diagnosed as COVID-19 between March 16, 2020, and June 15, 2020 were included. Diagnosis was confirmed by nasopharyngeal and oropharyngeal polymerase chain reaction (PCR). After diagnosis, the clinical and laboratory data of cases were extracted from patient records and database. Case characteristics including age, sex, dates of disease onset and diagnosis, family, and contact information were recorded. Clinical data, including the duration and severity of symptoms, were also collected. Laboratory parameters like biochemical tests and complete blood count, chest X-ray, and chest computerized tomography (CT) were recorded.

Finally, respiratory support (if present) and the duration of hospitalization were also determined. Data were entered by the participating pediatric infectious disease specialists.

### Definitions

Disease severity was defined based mainly on the clinical features and laboratory testing and, also if available, chest radiograph imaging and was classified as asymptomatic infection, mild, moderate, severe, or critical disease. The diagnostic criteria were as follows (9):

a- Asymptomatic infection: There was no clinical sign or symptom; the chest imaging results were normal, but the 2019-nCoV nucleic acid test result was positive.
b- Mild: There were symptoms of upper respiratory tract infection. Physical examination showed congestion of the pharynx and no auscultatory abnormalities.
c- Moderate: Patients with pneumonia, frequent fever, and cough were included; some had wheezing or rales. Cases who had no clinical signs and symptoms, but chest computed tomography showed subclinical lung lesions, were also included in this group.
d- Severe/critical: There was obvious hypoxemia and respiratory support (invasive or non-invasive) and intensive care are required.

### Sampling and Molecular Analysis

Both nasopharyngeal and oropharyngeal swabs were collected from suspected cases as recommended by Turkish COVID-19 guidelines (10). Collected respiratory samples taken from COVID-19 suspicious cases in hospitals were delivered to laboratories using viral transport media for respiratory viruses and following the cold chain rules. Viral transport media were prepared as 2 ml viral transport medium (VTM; MEM) supplemented with penicillin–streptomycin, and bovine serum albumin (BSA) was added, vortexed, homogenized, and transferred to 2 ml volumes of cryovial tubes. Nasopharyngeal aspirate, nasal wash water, throat wash water, transtracheal aspiration, and bronchoalveolar lavage samples were transferred directly to the cryovial tubes.

The process of extraction was done using the Bio-Speedy® Viral Nucleic Acid Extraction Kit (Cat No. BS-N-109, Bioeks R&D Ltd., Turkey) and PCR was made by the PCR run Bio-Speedy® Covidien-19 RT-qPCR Detection Kit (Cat No. BS SY–WC-305, Bioeks R&D Ltd., Turkey). After extraction and PCR, Bio-Rad CFX96 Touch™ (Bio-Rad Laboratories, Inc., USA) was included in the real-time PCR instrument. In the evaluation of the results, the proliferation curves obtained in the FAM/HEX channels were examined. Non-sigmoidal curves were recorded as negative. In cases where positive negative and internal control values met appropriate criteria, when Ct <40, the result was considered positive.

Samples before March 20 were analyzed in the National Molecular Microbiology Reference Laboratory; after March 20, all samples were tested in each center's microbiology laboratory individually by the same standard method.

### Statistical Analysis

The data were analyzed with SPSS (Statistical Package for the Social Sciences) 22.0 software (IBM SPSS Statistics, IBM Corporation). The numerical data were evaluated using visual (histograms, probability plots) and analytical methods (Kolmogorov–Smirnov test) to determine the distribution of normality. Median, range [interquartile range (IQR)], mean, standard deviation, number, and percentage were used as descriptive statistics, according to the normality of distribution.

Comparisons of categorical variables were made using the chi-square test. Comparisons of laboratory parameters between the two groups were made using *t*-test for independent samples or Mann–Whitney *U* test depending on the normality of distribution. The level of significance was considered as *p* < 0.05.

## Results

### Patient Demographics

There were 1,156 confirmed pediatric COVID-19 cases. In total, male cases constituted 50.3% (*n* = 582) and females constituted 49.7% (*n* = 574). The median age of the confirmed cases was 10.75 years (IQR: 4.5–14.6). Of the total cases, 90 were younger than 1 year of age (7.8%), 108 were 1–3 years of age (9.3%), 148 were 3–6 years of age (12.8%), 298 were 6–12 years of age (25.8%), 233 were 12–15 years of age (20.2%), and 268 cases were older than 15 years of age (23.2%). The age of 11 patients was not available. There were 893 patients who were symtomatic (77.2%). Among symptomatic cases, 81 (9.0%) were under 1 year of age and 217 of the cases (24.3%) were older than 15 years of age. The difference between age groups in case of symptom presence was statistically significant (*p* < 0.01).

Only 36 (3.1%) patients had a history of travel abroad (6 direct and 30 patients were housemates with people who had a history of travel). There was at least one COVID-19 case among family members of the 843 (75.2%) patients. Patient demographics and age distribution are shown in Table 1.

**Table 1:** Patient Demographics.

		**Total Cases**		**Symptomatic Cases**	
		***n***	**%**	***n***	**%**
Sex^*^	Male	582	50.3	457	51.2
	Female	574	49.7	436	48.8
Age groups^*^	0–1 year	90	7.8	81	9.0
	1–3 years	108	9.3	87	9.7
	3–6 years	148	12.8	117	13.1
	6–12 years	298	25.8	220	24.6
	12–15 years	233	20.2	169	18.9
	>15 years	268	23.2	217	24.3
History of travel abroad (direct or indirect) ^*^	–	1,104	96.9	870	97.1
	+	36	3.1	26	2.9
House-hold contact^*^	–	278	27.1	247	28.1
	+	843	72.9	631	71.9
	One case	273	37.3	213	38.5
	More than one case	457	62.7	340	61.5
Co-morbidities^*^	None	982	86.8	759	86.1
	Asthma/lung disease	47	4.1	41	4.6
	Malignancy	9	0.7	5	0.5
	Other immunosupressive situations	10	0.8	10	1.1
	Other	83	7.6	66	7.7

* *There are missing data in laboratory data, therefore sum is not equal to total number of patients*.

### Clinical Findings

The most common symptom of the patients at the first visit was fever (50.4%) (*n* = 583) for a median of 2 days (IQR: 1–3 days). Fever was median at 38.4°C (38.0–38.7°C). The second most common symptom was cough (*n* = 543, 46.9%). The other common symptoms were sore throat (*n* = 143, 12.4%), myalgia (*n* = 141, 12.2%), dyspnea (*n* = 118, 10.2%), diarrhea (*n* = 112, 9.7%), stomachache (*n* = 71, 6.1%), and nasal discharge (*n* = 63, 5.4%). Symptoms and their duration are summarized in Table 2.

**Table 2:** Clinical features.

		***N***	**%**	**Duration (median) (IQR)**
Fever^*^	+	583	50.4	2 (1–3)
	<38.3°C	120	–	
	38.3–38.5°C	62	–	
	≥38.6°C	83	–	
	–	530	45.8	
Cough^*^	+	543	46.9	3 (2–4)
	–	561	48.5	
Myalgia^*^	+	141	12.2	2 (2–3)
	–	977	84.5	
Dyspnea^*^	+	118	10.2	2 (1–3)
	–	1,011	87.4	
Stomachache^*^	+	71	6.1	3 (1-3)
	–	1,046	90.5	
Nasal discharge^*^	+	63	5.4	2.5 (2–3)
	–	1,062	91.8	
Diarrhea^*^	+	112	9.7	2 (1–3)
	–	1,017	87.9	
Sore throat^*^	+	143	12.4	2 (1–3)
	–	980	84.7	

* *There are missing data in laboratory data, therefore sum is not equal to total number of patients*.

Analyses of clinical symptoms based on sex revealed that fever was more common in males than in female cases (*p* < 0.01). Other symptoms did not differ according to sex (Tables 3, 4). Cough was more common in patients 6–12 years of age and fever was more common in patients older than 15 years of age (*p* < 0.01). Myalgia (43.6%) and diarrhea (21.3%) were more common in patients older than 15 years of age (*p* < 0.01). There was at least one family contact in 72.9% of the patients who had fever, in 65.8% of the patients who had diarrhea, and in 70.3% of the patients who had cough (*p* < 0.01). There was no difference between having one or more home-contact cases in case of symptoms and severity.

**Table 3:** Comparison of symptoms and laboratory parameters by means of sex and age groups among total cases.

			**SEX (*****n*****/%)**			**AGE GROUPS (year) (*****n*****/%)**						***p***
			**Male**	**Female**	***P***	**0–1**	**1–3**	**3–6**	**6–12**	**12–15**	**>15**	
Symptoms	Fever^*^	+	315 (56.6%)	268 (48.2%)	0.005	58 (69.9%)	76 (71.0%)	82 (57.3%)	145 (50.3%)	103 (45.8%)	118 (45.2%)	<0.001
		–	242 (43.4%)	288 (51.2%)		25 (30.1%)	31 (29.0%)	61 (42.7%)	143 (49.7%)	122 (54.2%)	143 (54.8%)	
	Cough^*^	+	271 (49.0%)	272 (49.3%)	0.9	50 (59.5%)	39 (37.5%)	67 (47.2%)	124 (42.4%)	115 (51.6%)	148 (58.5%)	<0.001
		–	282 (51.0%)	279 (50.7%)		34 (40.5%)	65 (62.5%)	75 (52.8%)	168 (57.6%)	108 (48.4%)	105 (41.5%)	
	Nasal discharge^*^	+	28 (5.0%)	35 (6.2%)	0.21	3 (3.5%)	15 (14.2%)	12 (8.2%)	9 (3.1%)	12 (5.3%)	12 (4.6%)	0.001
		–	536 (95.0%)	526 (93.8%)		82 (96.5%)	91 (85.8%)	134 (91.8%)	284 (96.9%)	215 (94.7%)	250 (95.4%)	
	Myalgia^*^	+	63 (11.2%)	78 (14.0%)	0.15	1 (1.3%)	4 (3.8%)	14 (9.7%)	24 (8.2%)	39 (17.1%)	59 (22.5%)	<0.001
		–	499 (88.8%)	478 (86.0%)		77 (98.7%)	102 (96.2%)	131 (90.3%)	269 (91.8%)	189 (82.9%)	203 (77.5%)	
	Stomachache^*^	+	36 (6.4%)	35 (6.3%)	0.51	3 (3.8%)	2 (1.9%)	11 (7.5%)	25 (8.5%)	13 (5.7%)	17 (6.5%)	0.19
		–	526 (93.6%)	520 (93.7%)		75 (96.2%)	104 (98.1%)	135 (92.5%)	268 (91.5%)	215 (94.3%)	243 (93.5%)	
	Diarrhea^*^	+	52 (9.2%)	60 (10.7%)	0.39	20 (23.3%)	13 (12%)	17 (13%)	15 (5.1%)	21 (9.6%)	23 (8.8%)	0.002
		–	515 (90.8%)	502 (89.3%)		66 (76.7%)	95 (88%)	127 (87%)	278 (94.9%)	206 (90.4%)	239 (91.2%)	
	Dyspnea^*^	+	57 (10.0%)	61 (10.9%)	0.64	20 (23%)	10 (9.3%)	9 (6.2%)	21 (7.2%)	16 (7%)	41 (15.6%)	<0.001
		–	511 (90.0%)	500 (89.1%)		67 (77%)	98 (90.7%)	137 (93.8%)	272 (92.8%)	211 (93%)	221 (84.4%)	
Lab	Anemia	+	41 (8.7%)	44 (9.2%)	0.77	26 (31%)	19 (20.7%)	13 (11.2%)	9 (3.8%)	9 (4.8%)	9 (4%)	<0.001
		–	431 (91.3%)	435 (90.8%)		58 (69%)	73 (79.3%)	103 (88.8%)	229 (96.2%)	179 (95.2%)	218 (96%)	
	Leucopenia	+	23 (4.8%)	44 (9.1%)	0.009	2 (2.3%)	2 (2.2%)	8 (6.8%)	13 (5.4%)	13 (6.9%)	28 (12.3%)	0.005
		–	452 (95.2%)	437 (90.9%)		85 (97.7%)	90 (97.8%)	109 (93.2%)	226 (94.6%)	175 (93.1%)	199 (87.7%)	
	Lypmhopenia	+	95 (20%)	143 (29.9%)	<0.001	5 (5.7%)	4 (4.4%)	20 (17.2%)	54 (22.8%)	55 (29.3%)	99 (43.6%)	<0.001
		–	379 (80%)	336 (70.1%)		82 (94.3%)	88 (95.7%)	96 (82.8%)	183 (77.2%)	133 (70.7%)	128 (56.4%)	
	Thrombocytopenia	+	34 (7.2%)	24 (5%)	0.39	3 (3.4%)	3 (3.3%)	7 (6%)	16 (6.7%)	8 (4.3%)	21 (9.3%)	0.45
		–	441 (92.8%)	457 (95%)		84 (96.6%)	89 (96.7%)	110 (94%)	223 (93.3%)	180 (95.7%)	206 (90.7%)	
	CRP	High	191 (39.9%)	142 (29.6%)	0.001	29 (33.7%)	33 (35.9%)	43 (37.4%)	93 (38.1%)	58 (30.9%)	75 (32.9%)	0.74
		Normal	288 (60.1%)	338 (70.4%)		57 (66.3%)	59 (64.1%)	72 (62.6%)	151 (61.9%)	130 (69.1%)	153 (67.1%)	
	AST	High	72 (16.7%)	53 (12.2%)	0.06	48 (60%)	30 (34.9%)	13 (11.9%)	22 (10.2%)	5 (3.1%)	7 (3.4%)	<0.001
		Normal	358 (83.3%)	380 (87.8%)		32 (40%)	56 (65.1%)	96 (88.1%)	194 (88.8%)	156 (96.9%)	198 (96.6%)	
	ALT	High	36 (7.7%)	16 (3.4%)	0.004	6 (6.9%)	6 (6.6%)	2 (1.8%)	18 (7.7%)	8 (4.3%)	12 (5.4%)	0.25
		Normal	429 (92.3%)	457 (96.6%)		81 (93.1%)	85 (93.4%)	110 (98.2%)	216 (92.3%)	177 (95.7%)	211 (94.6%)	
	CK level	High	99 (26.2%)	59 (14.9%)	<0.001	33 (49.3%)	32 (42.7%)	18 (19.8%)	25 (13.2%)	20 (13.1%)	30 (15.6%)	<0.001
		Normal	279 (73.8%)	337 (85.1%)		34 (50.7%)	43 (57.3%)	73 (80.2%)	165 (86.8%)	133 (86.9%)	162 (84.4%)	
	Troponin level	High	9 (2.6%)	7 (1.9%)	0.35	5 (8.3%)	2 (3.1%)	1 (1.3%)	5 (2.8%)	0 (0%)	3 (1.7%)	0.01
		Normal	335 (97.4%)	356 (98.1%)		55 (91.7%)	62 (96.9%)	79 (98.8%)	172 (97.2%)	149 (100%)	172 (98.3%)	

** *There are missing data in laboratory data, therefore sum is not equal to total number of patients*.

* *The age of 6 patients was not known*.

**Table 4:** Comparison of symptoms and laboratory parameters by means of sex and age groups among symptomatic cases^*^.

			**SEX (*****n*****/%)**			**AGE GROUPS (month) (*****n*****/%)**						***p***
			**Male**	**Female**	***p***	**0–1**	**1–3**	**3–6**	**6–12**	**12–15**	**>15**	
Symptoms	Fever	+	315 (71.6%)	268 (62.9%)	0.006	58 (78.4%)	76 (87.4%)	82 (72.6%)	145 (68.1%)	103 (62%)	117 (55.5%)	<0.001
		–	125 (28.4%)	158 (37.1%)		16 (21.6%)	11 (12.6%)	31 (27.4%)	68 (31.9%)	63 (38%)	94 (44.5%)	
	Cough	+	271 (62.3%)	272 (64.5%)	0.53	50 (66.7%)	39 (47%)	67 (59.8%)	124 (57.4%)	115 (70.1%)	148 (72.2%)	<0.001
		–	164 (37.7%)	150 (35.5%)		25 (33.3%)	44 (53%)	45 (40.2%)	92 (42.6%)	49 (29.9%)	57 (27.8%)	
	Nasal discharge	+	28 (6.3%)	35 (8.1%)	0.29	3 (3.9%)	15 (17.6%)	12 (10.3%)	9 (4.1%)	12 (7.1%)	12 (5.6%)	0.001
		–	418 (93.7%)	398 (91.9%)		74 (96.1%)	70 (82.4%)	104 (89.7%)	209 (95.9%)	156 (92.9%)	201 (94.4%)	
	Myalgia	+	63 (14.2%)	78 (18.3%)	0.09	1 (1.4%)	4 (4.7%)	14 (12.2%)	24 (11%)	39 (23.1%)	59 (27.7%)	<0.001
		–	381 (85.8%)	349 (81.7%)		68 (98.6%)	81 (95.3%)	101 (87.8%)	194 (89%)	130 (76.9%)	154 (72.3%)	
	Stomach-ache	+	36 (8.1%)	35 (8.2%)	0.94	3 (4.3%)	2 (2.4%)	11 (9.5%)	25 (11.5%)	13 (7.7%)	17 (8.1%)	0.08
		–	408 (91.9%)	391 (91.8%)		66 (95.7%)	83 (97.6%)	105 (90.5%)	193 (88.5%)	156 (92.3%)	194 (91.9%)	
	Diarrhea	+	52 (11.6%)	60 (13.9%)	0.30	20 (26%)	13 (1.9)	19 (16.4%)	15 (6.9%)	22 (13%)	23 (10.8%)	0.04
		–	397 (88.4%)	373 (86.1%)		57 (74%)	74 (85.1%)	97 (83.6%)	203 (93.1%)	147 (87%)	190 (88.2%)	
	Dyspnea	+	57 (12.7%)	61 (14.2%)	0.51	20 (25.6%)	10 (11.5%)	9 (7.8%)	21 (9.6%)	16 (9.5%)	41 (19.2%)	<0.001
		–	393 (87.3%)	371 (85.8%)		58 (74.4%)	77 (88.5%)	107 (92.2%)	197 (90.4%)	152 (90.5%)	172 (80.8%)	
Lab	Anemia	+	35 (9.2%)	35 (9.4%)	0.93	25 (33.3%)	14 (18.7%)	11 (11.8%)	5 (2.8%)	8 (5.7%)	7 (3.7%)	<0.001
		–	346 (90.8%)	339 (90.6%)		50 (66.7%)	61 (81.3%)	82 (88.2%)	174 (97.2%)	133 (94.3%)	183 (96.3%)	
	Leukopenia	+	20 (5.2%)	35 (9.3%)	0.02	2 (2.6%)	2 (2.7%)	6 (6.4%)	12 (6.7%)	8 (5.7%)	25 (13.2%)	0.01
		–	364 (94.8%)	341 (90.7%)		76 (97.4%)	73 (97.3%)	88 (93.6%)	168 (93.3%)	133 (94.3%)	165 (86.8%)	
	Lypmhopenia	+	80 (20.9%)	122 (32.6%)	<0.001	5 (6.4%)	4 (5.3%)	18 (19.4%)	44 (24.7%)	46 (32.6%)	85 (44.7%)	<0.001
		–	303 (79.1%)	252 (67.4%)		73 (93.6%)	71 (94.7%)	75 (80.6%)	134 (75.3%)	95 (67.4%)	105 (55.3%)	
	Thrombocytopenia	+	26 (6.8%)	21 (5.6%)	0.49	2 (2.6%)	2 (2.7%)	5 (5.3%)	14 (7.8%)	8 (5.7%)	16 (8.4%)	0.31
		–	358 (93.2%)	355 (94.4%)		76 (97.4%)	73 (97.3%)	89 (94.7%)	166 (92.2%)	133 (94.3%)	174 (91.6%)	
	CRP	High	174 (45.3%)	121 (32.3%)	<0.001	28 (36.4%)	31 (41.3%)	41 (44.1%)	78 (43.3%)	46 (32.6%)	69 (36.1%)	0.36
		Normal	210 (54.7%)	254 (67.7%)		49 (63.6%)	44 (58.7%)	52 (55.9%)	102 (56.7%)	95 (67.4%)	122 (63.9%)	
	CK level	High	83 (27.1%)	55 (17.5%)	0.004	31 (50.8%)	31 (50%)	13 (18.1%)	20 (13.4%)	18 (15.5%)	25 (15.7%)	<0.001
		Normal	224 (73%)	259 (82.5%)		30 (49.2%)	31 (50%)	59 (81.9%)	129 (86.6%)	98 (84.5%)	134 (84.3%)	
	Troponin level	High	7 (2.5%)	7 (2.4%)	0.93	5 (8.9%)	2 (3.8%)	1 (1.6%)	4 (3.0%)	0 (0%)	2 (1.3%)	0.01
		Normal	270 (97.5%)	283 (97.6%)		51 (91.1%)	51 (96.2%)	61 (98.4%)	127 (96.9%)	113 (100%)	148 (98.7%)	
	AST	High	65 (18.4%)	289 (81.6%)	0.07	43 (60.6%)	28 (38.9%)	11 (12.5%)	19 (11.4%)	4 (3.2%)	6 (3.4%)	<0.001
		Normal	46 (13.3%)	301 (86.7%)		28 (39.4%)	44 (61.1%)	77 (87.5%)	147 (88.6%)	122 (96.8%)	170 (96.6%)	
	ALT	High	29 (7.7%)	14 (3.8%)	0.02	5 (6.4%)	5 (6.7%)	2 (2.2%)	16 (9.1%)	5 (3.6%)	10 (5.3%)	0.14
		Normal	346 (92.3%)	358 (96.2%)		73 (93.6%)	70 (93.3%)	87 (97.8%)	160 (90.9%)	134 (96.4%)	178 (94.7%)	

* *There are missing data in some variables, therefore sum is not equal to total number of patients*.

Of the total cases, 149 (12.9%) had underlying diseases, and 56% of the patients who had severe disease had an underlying condition (*p* < 0.01). The need for hospitalization did not differ between patients who had an underlying condition and those who do not have (*p* = 0.38), but the need for intensive care was higher in patients who had an underlying condition (*p* < 0.01). Forty-seven (31.5%) of the cases having underlying conditions had asthma or lung disease (38 of them had asthma). The prevalence of asthma was 3.2% in this study group. Cough was observed mainly in asthmatic patients (80.0%) and 87.6% of patients with asthma were symptomatic (*p* = 0.016). However, having asthma was not significantly related to disease severity (*p* = 0.62). There were 19 immunocompromised patients (because of malignancies or other conditions) and fever was present in 78.6% of them, but this relationship was not statistically significant (*p* = 0.36).

When patients were classified according to disease severity, 263 (22.7%) patients were asymptomatic, 668 (57.7%) patients had mild disease, 209 (18.1%) had moderate disease, and 16 (1.5%) cases had severe disease. There was no statistically significant difference between girls and boys in case of symptom presence (*p* = 0.108), but when asymptomatic cases were excluded, it was observed that girls tend to have the disease more seriously (*p* = 0.04) (Table 5). Nineteen (1.6%) patients needed intensive care during follow-up. Eleven of them had underlying diseases and two of the underlying diseases were immune-suppressive conditions. Five (0.4%) patients needed mechanical ventilation and two patients died (0.1%).

**Table 5:** Comparison of groups due to severity^*^.

			**Aymptomatic**	**Symptomatic**				***p***
				**Mild**	**Moderate**	**Severe**	***p***	
SEX	Female		130 (23%)	311 (55%)	113 (20.0%)	11 (1.9%)	0.04	0.32
	Male		118 (20.6%)	355 (61.8%)	96 (16.7%)	5 (0.9%)		
Age Groups	0–1 yr		9 (3.5%)	54 (58.3%)	23 (27.4%)	4 (4.8%)	<0.001	0.006
	1–3 yrs		21 (8.3%)	70 (63.0%)	15 (14.0%)	2 (2.0%)		
	3–6 yrs		31 (12.2%)	99 (63.9%)	16 (12.0%)	2 (1.5%)		
	6–12 yrs		78 (30.7%)	181 (58.0%)	35 (12.6%)	4 (1.5%)		
	12–15 yrs		64 (25.2%)	124 (52.2%)	43 (20.9%)	2 (1.0%)		
	>15 yrs		51 (20.1%)	139 (50.4%)	76 (29.8%)	2 (0.8%)		
Symptoms	Fever	– +	–	215 (76.0%) 438 (75.1%)	65 (23.0%) 132 (22.6%)	3 (1.1%) 13 (2.2%)	0.48	
	Cough	– +	–	265 (84.4%) 392 (72.2%)	41 (13.1%) 143 (26.3%)	8 (2.5%) 8 (1.5%)	0.007	
	Myalgia	– +	–	558 (76.4%) 101 (71.6%)	161 (22.1%) 38 (27.0%)	11 (1.5%) 2 (1.4%)	0.44	
	Diarrhea	– +	–	581 (75.5%) 82 (73.2%)	177 (23.0%) 26 (23.2%)	12 (1.6%) 4 (3.6%)	0.32	
	Dyspnea	– +	–	610 (79.8%) 54 (45.8%)	150 (19.6%) 52 (44.1%)	4 (0.5%) 12 (10.2%)	<0.001	
Radiology	X-Ray	Finding – Finding +	163 (87.6%) 22 (12.4%)	523 (91.4%) 79 (34.5%)	43 (7.5%) 143 (62.4%)	6 (1.1%) 7 (3.1%)	<0.001	<0.001
	CT	Finding – Finding +	45 (81.8%) 10 (18.2%)	227 (98.7%) 1 (0.5%)	2 (0.9%) 191 (95.5%)	1 (0.4%) 8 (4.0%)	<0.001	<0.001

* *There are missing data in some variables, therefore sum is not equal to total number of patients*.

Asymptomatic disease was higher in the 6–12 year age group (36.8%) and moderate disease was higher in cases who were older than 15 years of age (*p* < 0.01). Cough, dyspnea, and fever were significantly related to severe disease (*p* < 0.01), whereas myalgia, diarrhea, rhinorrhea, sore throat, and stomachache were not related to severe disease (*p* > 0.05) (Table 5).

### Laboratory Findings

In 85 of the total cases (8.9%), 70 of the symptomatic cases had anemia (9.3%). In 67 of the total cases (7.0%), 55 of the symptomatic cases (7.3%) had leukopenia. Lymphopenia was seen in a total of 238 patients (20.5%) and 202 of them were symptomatic. There was a statistically significant difference between symptomatic and asymptomatic cases in case of leukocyte count (*p* = 0.009) but not in case of lymphocyte count (*p* = 0.09) (Table 5). There were 58 cases among the total cases who had thrombocytopenia (5.0%) as well as 47 cases among symptomatic cases (5.3%). Three hundred and thirty-three cases among the total cases had high C-reactive protein (CRP) levels (34.7%), whereas among symptomatic cases, 295 had normal levels (33.0%). Patients who had symptoms had higher CRP levels than those who were asymptomatic (*p* < 0.01) (Table 5). Alanine transaminase (ALT) level of 52 cases was high among the total cases (5.5%) as well as of 43 cases (5.8%) of the symptomatic cases. One hundred and twenty-five cases (14.5%) of the total study population and 110 (15.7%) of the symptomatic cases had high aspartate transaminase (AST) levels (*p* = 0.02). Both ALT and AST levels did not differ between symptomatic and asymptomatic cases. The laboratory examination of the patients is summarized in Table 6.

**Table 6:** Laboratory findings.

	**Total Cases**	**Symptomatic Cases**	**Asymptomatic Cases**	***p***
	**Median (IQR)**	**Median (IQR)**	**Median (IQR)**	
Hemoglobin (g/dL)	12.9 (12-14)	12.8 (11.9–13.9)	13.0 (12.1–14.0)	0.09
Leukocyte count (/uL)	6,615 (5,222–9,187)	6,780 (5,275–9,560)	6,219 (5,157–8,260)	0.009
Lymphocyte count (/uL)	2,200 (1,490–3,285)	2,100 (1,435–3,300)	2,320 (1,700–3,235)	0.09
Thrombocyte (/uL)	248,000 (207,000–306,750)	245,000 (204,000–306,000)	262,500 (219,000–312,000)	0.13
C-reactive protein (mg/L)	2.73 (2.73–7.78)	3.1 (1.0–10.7)	1.1 (0.7–3.0)	<0.001
Aspartate transaminase (U/L)	28 (21–37)	27 (20.7–35)	28 (22.0–33.5)	0.6
Alanin transaminase (U/L)	17 (13–24)	15 (12–21)	17 (13.0–24.0)	0.9
Creatin kinase (U/L)	89 (65–121)	89 (63–123)	91 (68–115)	0.8
Troponin (ng/L)	0.1 (0.1–2.3)	0.1 (0.1–2.7)	0.1 (0.1–2.3)	0.6

Leukopenia and lymphopenia were more common in female cases (*p* < 0.01). CRP levels were significantly higher in male patients (*p* < 0.01). According to age classification, leukopenia (12.6%) and lymphopenia (44.1%) were more common in patients who were ≥15 years of age (*p* < 0.01) (Table 4).

Chest X-ray was performed in 988 patients. Among these patients, 736 (74.5%) had normal results, whereas 252 (25.5%) had at least one pathologic finding. CT was performed in 485 patients. Of these patients, 275 (56.7%) had normal results. The most common finding was one or more ground-glass opacities in 126 (43.2%) patients. Computerized tomography was performed in 269 patients (38.8%) whose chest X-ray was normal, and there were pathologic findings on CT in 48 (17.8%) of the patients (*p* < 0.01) (Table 5).

## Discussion

To the best of our knowledge, this is one of the largest pediatric data reported about COVID-19, with 1,156 cases. Supporting the current literature, male cases were slightly more than female cases in our study (11–14).

According to the age distribution of patients, the majority of patients in our study were between 6 and 12 years of age, which constituted 25.8% of the total confirmed cases. Interestingly, this age group is composed of school-age children, although school education was interrupted just 2 days after the first case reported in Turkey. This finding was also similar to the study of Lu et al. (11). In a review by Ding et al., the majority of pediatric patients with COVID-19 were older than 5 years of age (13). These findings might be due to the difficulties in providing personal hygiene procedures and infection control precautions, ideally in this age group. However, as the ages ranged from 1 day to 18 years, it was shown that all ages of childhood were susceptible to 2019-nCoV. Neither difference between sex groups nor age groups can be explained by the susceptibility of these groups to COVID-19 infection because no sufficient data is available yet. Further studies should be warranted about this topic.

There were 75.2% of the patients who had household contacts; no cases caused by schoolmates were reported. According to the report of Hoang et al., 75.6% of pediatric patients were exposed to a family member who was diagnosed with COVID-19 (14). This ratio is as high as 86.4% in another review by Ding et al. (13).

Another important point is that there were only 36 cases who had a direct/indirect history of travel. This finding emphasizes that quarantine after traveling abroad is important for the prevention of the spread of the virus. On the other hand, home contact and exposure still remain an important issue for the spread of COVID-19 in children (11, 12).

Xia et al. indicated that patients with a history of congenital or acquired diseases would have a greater susceptibility to COVID-19 (15). According to the CDC-MMWR, 23% of pediatric patients had underlying conditions. The most common underlying conditions were chronic lung disease (including asthma) followed by cardiovascular disease and immunosuppression (16). In another report, 6.1% of all the included children had underlying diseases (13). According to another report, the most common underlying medical condition was immunosuppression history of a respiratory or cardiac condition (65%) (14). Similarly, our findings also support that having an underlying disease was a risk factor for disease severity and the need for intensive care (*p* < 0.01) but interestingly not for hospitalization (*p* = 0.37).

There were 12.9% of our patients who had underlying conditions; the most common were asthma and lung disease. According to a review, only one report was found describing asthma/recurrent wheezing as a potential risk factor for COVID-19 in children (17), but none of the largest epidemiological studies including children with COVID-19 reported clinical findings or underlying characteristics to help assess whether asthma—or other chronic lung diseases—constitutes a risk factor for SARS-CoV-2 infection or COVID-19 severity. Our data also do not conclude that asthma increases the risk for COVID-19, but may draw attention to this issue and shed light on further studies. Besides, despite the rate of 3.2%, the fact that asthmatic patients generally have symptomatic but not severe COVID-19 is a new and important finding in the literature and must be supported by future prospective studies.

Two hundred and sixty-three (22.7%) patients were asymptomatic, whereas 893 (77.3%) were symptomatic. Among symptomatic patients, 668 (74.8%) patients had mild disease, 209 (23.4%) had moderate disease, and 16 (1.8%) had severe disease. Dong et al. reported that 43.1% of the confirmed cases were asymptomatic, and 12.9% had mild disease (12). In the systematic review of Hoang et al., 19.2% of the cases were asymptomatic (14). According to the study of Lu et al., patients who were asymptomatic revealed 15.8% and who had mild disease revealed 19.3% of confirmed patients (11). The findings of our study were similar to the literature in general as children with COVID-19 tend to be asymptomatic or just have mild symptoms.

Similar to the current literature, the most common symptoms were fever and cough in our study (11–13, 16). In the report of Hoang et al., the common clinical manifestations included fever (60%), whereas nasal congestion and rhinorrhea were the second most common symptoms (28%) (14). In contrast, cough (24%) was not reported as high as in our study. In addition, although myalgia is known as one of the leading symptoms of COVID-19 in children, frequency information is not common in the literature (15, 18–20), but myalgia was reported as 12.2% of the total cases and 15.7% of the symptomatic cases in our study, which is a significant proportion. Besides, it was significantly high in patients who were older than 15 years (*p* < 0.01).

There are many reports about the laboratory findings in pediatric COVID-19 patients, but generally, no classification by age groups or sex or disease severity was given. In a pediatric review, according to laboratory findings, leukopenia and leukocytosis were present in 7.3 and 10.7% of the cases, respectively (11, 15, 18–24). According to another review, the most frequent abnormal laboratory findings in pediatric patients were leukopenia/lymphopenia and increased creatine kinase (13). Lymphopenia and lymphocytosis were present in 21 and 5% of the cases, respectively; thrombocytopenia was found in only 4% of the cases (13). According to our findings, leukopenia was observed in 5.7% of the cases, and lymphopenia was observed in 20.5% of the total cases. It seems likely that COVID-19 has an effect in the direction of cytopenia, like other viral infections, especially in adolescents.

Chest X-ray was performed in 988 patients. Among these patients, 736 (74.5%) had normal results, whereas 252 (25.5%) had at least one pathologic finding. CT was performed in 485 patients. Of these patients, 275 (56.7%) had normal results. Computerized tomography was performed in 269 patients (38.8%) whose chest X-ray was normal, and there were pathologic findings on CT in 48 (17.8%) of the patients (*p* < 0.01). These findings support that CT does not need to be used as frequently as in adults because of the less severe character of the disease in children. It makes more sense to consider computerized tomography in moderate or severe patients, to avoid exposure of this sensitive population to radiation.

One of the limitations of this study is the retrospective nature of the study. Secondly, there were missing data especially in case of symptomatology and diagnostic tests and this caused difficulty in standardizing the results for the whole study population.

## Conclusion

To the best of our knowledge, this is the largest pediatric data about confirmed COVID-19 cases in the current literature from Turkey and also the largest globally. Similar to the current literature, according to our study, children seem to be less frequently infected. However, current evidence is still limited regarding children with the virus infecting other people.

Firstly, it is remarked that myalgia is one of the major symptoms of COVID-19, which is present in up to 16.2% of the symptomatic cases, especially in adolescents. According to our study, asthma seems to be a significant underlying disease for COVID-19 patients, but future studies are warranted to prove it as a risk factor. Finally, we state that computerized tomography is mostly normal except for severe cases actually, and it is important to use it in selected patients to avoid unnecessary exposure to radiation. As we all know, huge data are changing every day, and future studies are needed to support our findings in the field of pediatrics.

## Data Availability Statement

The original contributions presented in the study are included in the article/supplementary material, further inquiries can be directed to the corresponding author/s.

## Ethics Statement

The studies involving human participants were reviewed and approved by the Turkish Republic, Ministry of Health.

## Author Contributions

All authors contributed to the study equally by means of conception, protocol development, data collection, data interpretation, and critical revision of the manuscript.

## Conflict of Interest

The authors declare that the research was conducted in the absence of any commercial or financial relationships that could be construed as a potential conflict of interest.

## References

[1] WHO Timeline - COVID-19. Available online at: https://www.who.int/news-room/detail/27-04-2020-who-timeline—covid-19 (accessed May 1, 2020).

[2] WHO Director-General's Statement on IHR Emergency Committee on Novel Coronavirus (2019-nCoV). Available online at: https://www.who.int/dg/speeches/detail/who-director-general-s-statement-on-ihr-emergency-committee-on-novel-coronavirus-(2019-ncov) (accessed May 1, 2020).

[3] Weekly Epidemiological Update - 27 January 2021. Available online at: https://www.who.int/publications/m/item/weekly-epidemiological-update-−27-january-2021 (accessed February 2, 2021).

[4] Lee PI Hu YL Chen PY Huang YC Hsueh PR. Are children less susceptible to COVID-19? J Microbiol Immunol Infect. (2020) 53:371–2. 10.1016/j.jmii.2020.02.011 32147409 PMC7102573

[5] Lanziotti VS de Souza DC Marques ETA. Coronavirus disease 2019: understanding immunopathogenesis is the “Holy Grail” to explain why children have less severe acute disease. Pediatr Crit Care Med. (2020) 21:1022–3. 10.1097/PCC.0000000000002513 32618678 PMC7340132

[6] Riphagen S Gomez X Gonzalez-Martinez C Wilkinson N Theocharis P. Hyperinflammatory shock in children during COVID-19 pandemic. Lancet. (2020) 395:1607–8. 10.1016/S0140-6736(20)31094-1 32386565 PMC7204765

[7] Wei M Yuan J Liu Y Fu T Yu X Zhang Z-J. Novel coronavirus infection in hospitalized infants under 1 year of age in China. JAMA. (2020) 323:1313–4. 10.1001/jama.2020.2131 32058570 PMC7042807

[8] Cao Q Chen YC Chen CL Chiu CH. SARS-CoV-2 infection in children: transmission dynamics and clinical characteristics. J Formos Med Assoc. (2020) 119:670–3. 10.1016/j.jfma.2020.02.009 32139299 PMC7126646

[9] Society of Pediatrics Chinese Medical Association; Editorial Board Chinese Journal of Pediatrics. Recommendations for the diagnosis, prevention, and control of the 2019 novel coronavirus infection in children (first interim edition). Zhonghua Er Ke Za Zhi. (2020) 58:E004. 10.3760/cma.j.issn.0578-1310.2020.0004 32035429

[10] COVID-19: Novel Corona Virus Disease Guideline. Turkish Republic, Ministry of Health. Available online at: https://covid19bilgi.saglik.gov.tr/tr/covid-19-rehberi.html (accessed June 25, 2020).

[11] Lu X Zhang L Du H Zhang J Li YY Qu J. SARS-CoV-2 infection in children. N Engl J Med. (2020) 382:1663–5. 10.1056/NEJMc2005073 32187458 PMC7121177

[12] Dong Y Mo X Hu Y Qi X Jiang F Jiang Z. Epidemiology of COVID-19 among children in China. Pediatrics. (2020) 145:e20200702. 10.1542/peds.2020-0702 32179660

[13] Ding Y Yan H Guo W. Clinical characteristics of children with COVID-19: a meta-analysis. Front Pediatr. (2020) 8:431. 10.3389/fped.2020.00431 32719759 PMC7350605

[14] Hoang A Chorath K Moreira A Evans M Burmeister-Morton F Burmeister F. COVID-19 in 7780 pediatric patients: a systematic review. EClinicalMedicine. (2020) 24:100433. 10.1016/j.eclinm.2020.100433 32766542 PMC7318942

[15] Xia W Shao J Guo Y Peng X Li Z Hu D. Clinical and CT features in pediatric patients with COVID-19 infection: different points from adults. Pediatr Pulmonol. (2020) 55:1169–74. 10.1002/l.24718 32134205 PMC7168071

[16] CDC COVID-19 Response Team. Coronavirus disease 2019 in Children- United States, February 12- April 2, 2020. MMWR Morb Mortal Wkly Rep. (2020) 69:422–6. 10.15585/mmwr.mm6914e4 32271728 PMC7147903

[17] Castro-Rodriguez JA Forno E. Asthma and COVID-19 in children: a systematic review and call for data. Pediatr Pulmonol. (2020) 55:2412–28. 10.1002/ppul.24909 32558360 PMC7323291

[18] Mustafa NM A Selim L. Characterisation of COVID-19 pandemic in paediatric age group: a systematic review and meta-analysis. J Clin Virol. (2020) 128:104395. 10.1016/j.jcv.2020.104395 32417675 PMC7207144

[19] Jiehao C Jin X Daojiong L Zhi Y Lei X Zhenghai Q. A Case Series of children with 2019 novel coronavirus infection: clinical and epidemiological features. Clin Infect Dis. (2020) 71:1547–51. 10.1093/cid/ciaa198 32112072 PMC7108143

[20] Hasan A Mehmood N Fergie J. Coronavirus disease (COVID-19) and pediatric patients: a review of epidemiology, symptomatology, laboratory and imaging results to guide the development of a management algorithm. Cureus. (2020) 12:e7485. 10.7759/cureus.7485 32257728 PMC7123290

[21] Liu W Zhang Q Chen J Xiang R Song H Shu S. Detection of Covid-19 in children in early January 2020 in Wuhan, China. N Engl J Med. (2020) 382:1370–1. 32163697 10.1056/NEJMc2003717 PMC7121643

[22] Sun D Li H Lu XX Xiao H Ren J Zhang FR. Clinical features of severe pediatric patients with coronavirus disease 2019 in Wuhan: a single center's observational study. World J Pediatr. (2020) 16:251–9. 10.1007/s12519-020-00354-4 32193831 PMC7091225

[23] Ji LN Chao S Wang YJ Li XJ Mu XD Lin MG. Clinical features of pediatric patients with COVID-19: a report of two family cluster cases. World J Pediatr. (2020) 16:267–70. 10.1007/s12519-020-00356-2 32180140 PMC7091281

[24] Soltani J Sedighi I Shalchi Z Sami G Moradveisi B Nahidi S. Pediatric coronavirus disease 2019 (COVID-19): an insight from west of Iran. North Clin Istanb. (2020) 7:284–91. 10.14744/nci.2020.90277 32478302 PMC7251275

